# Supplementary material for: Ribo-On and Ribo-Off tools using a self-cleaving ribozyme allow manipulation of endogenous gene expression in C. elegans
Source: Commun Biol. 2023 Aug 4;6:816. doi: 10.1038/s42003-023-05184-4 (PMC10403566; doi:10.1038/s42003-023-05184-4)
Supplement: Supplementary file 2 — Supplementary Information [file 42003_2023_5184_MOESM2_ESM.pdf]

Supplementary Information

Ribo-On and Ribo-Off tools using a self-cleaving ribozyme allow manipulation of endogenous gene expression in *C. elegans*

Jie Fang[1,2,3#], Jie Wang[1,2#], Yuzhi Wang[1,2], Xiaofan Liu[1,2], Baohui Chen[3,4,5,6*] & Wei Zou[1,2*]

This file contains:

-Supplementary Figure 1
-Supplementary Figure 2
-Supplementary Figure 3
-Supplementary Figure 4
-Supplementary Figure 5
-Supplementary Figure 6
-Supplementary Table 1
-Supplementary Table 2
-Supplementary Table 3
-Supplementary Table 4
-Supplementary Table 5

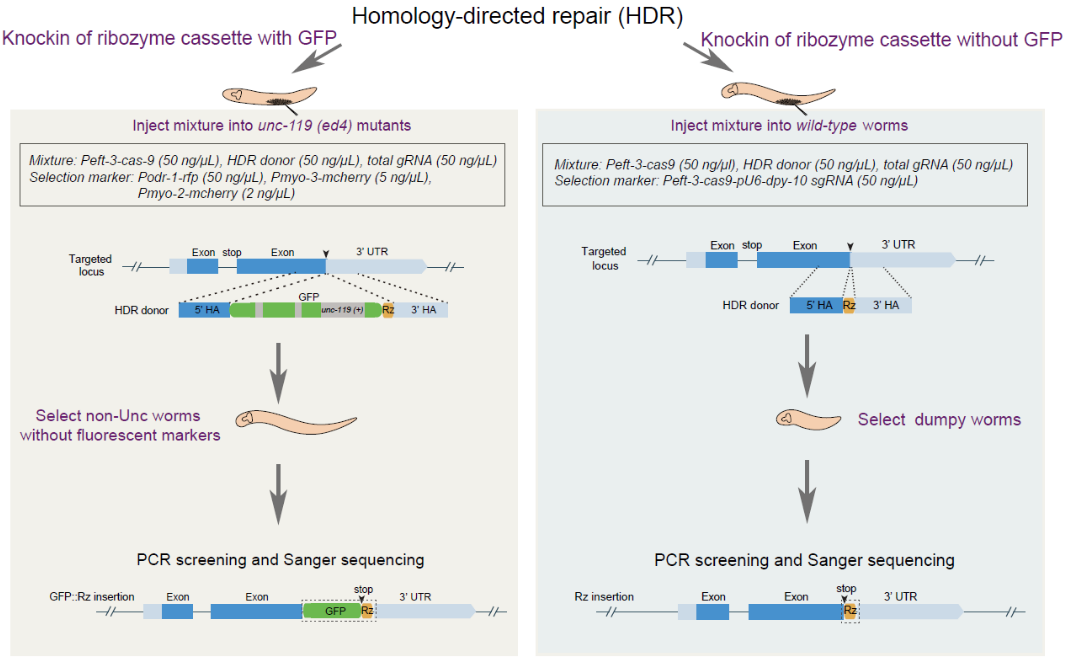

Supplementary Fig. 1

Strategies to generate ribozyme-knockin animals via CRISPR-mediated homology-directed repair.

To achieve a high knock-in efficiency of ribozyme cassettes at the endogenous locus, two strategies were devised: *unc-119*-based positive selection (left) and *dpy-10*-based co-CRISPR (right). To confirm that all animals carried the ribozyme cassette insertion in both targeted alleles, PCR-based genotyping and DNA sequencing were performed on the progeny resulting from self-fertilization.

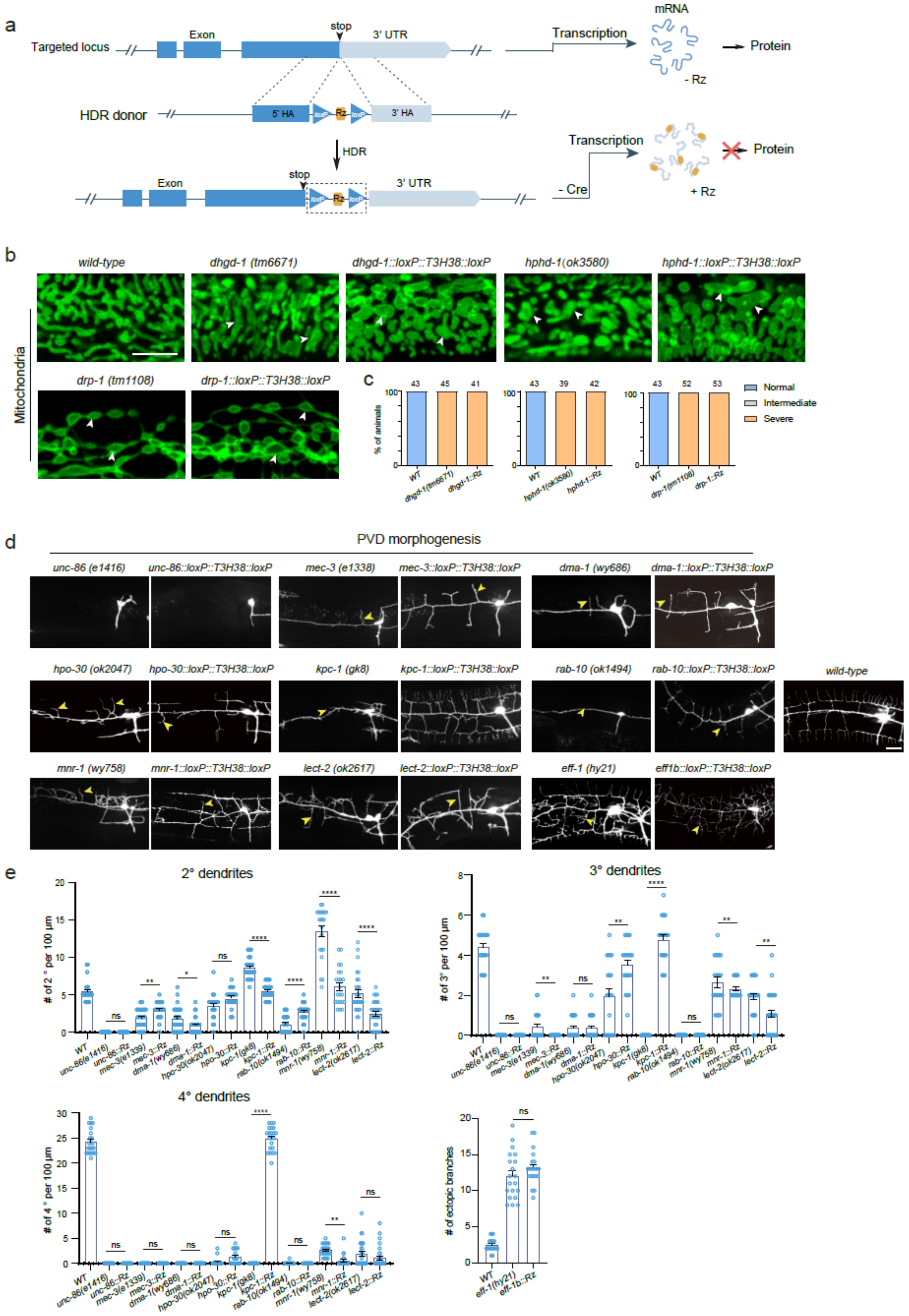
a
Targeted locus
Exon
stop
3' UTR
Transcription
mRNA
Protein
- Rz
HDR donor
5' HA
loxP
Rz
3' HA
HDR
+ Rz
- Cre
b
wild-type
dhgd-1 (tm6671)
dhgd-1::loxP::T3H38::loxP
hphd-1(ok3580)
hphd-1::loxP::T3H38::loxP
Mitochondria
drp-1 (tm1108)
drp-1::loxP::T3H38::loxP
c
% of animals
Normal
Intermediate
Severe
d
PVD morphogenesis
unc-86 (e1416)
unc-86::loxP::T3H38::loxP
mec-3 (e1338)
mec-3::loxP::T3H38::loxP
dma-1 (wy686)
dma-1::loxP::T3H38::loxP
hpo-30 (ok2047)
hpo-30::loxP::T3H38::loxP
kpc-1 (gk8)
kpc-1::loxP::T3H38::loxP
rab-10 (ok1494)
rab-10::loxP::T3H38::loxP
wild-type
mnr-1 (wy758)
mnr-1::loxP::T3H38::loxP
lect-2 (ok2617)
lect-2::loxP::T3H38::loxP
eff-1 (hy21)
eff1b::loxP::T3H38::loxP
e
2° dendrites
# of 2° per 100 μm
3° dendrites
# of 3° per 100 μm
4° dendrites
# of 4° per 100 μm
# of ectopic branches
WT
eff-1(hy21)
eff-1b::Rz

Supplementary Fig. 2

Additional endogenous genes inactivated by ribozyme knockin.

a Schematic illustration of using CRISPR/Cas9-mediated homologous recombination to insert the *loxP::Rz::loxP* or *gfp::loxP::Rz::loxP* cassette into the C-terminal of the endogenous target gene's locus, and the outcome of protein expression with or without Cre expression. **b** Confocal images showing mitochondrial morphology in wild-type and mutant animals. *dhgd-(tm6671)*, *hphd-1(ok3580) and drp-1(tm1108)* are strong loss-of-function or null alleles, causing enlarged mitochondrial phenotypes and are included for comparisons. Arrowheads: enlarged mitochondria. Scale bars: 5 μm. **c** Quantifications of the present of mitochondria-normal and -defective animals of day 1 adults in different genetic backgrounds as shown in (**b**). n⩾39 worms. **d** Confocal images showing the morphology of PVD dendrites. *unc-86(e1416)*, *mec-3(e1338)*, *dma-1(wy686)*, *hpo-30(ok2047)*, *kpc-1(gk8)*, *rab-10(ok1494)*, *mnr-1(wy758)*, *lect-2(ok2617)* and *eff-1(hy21)* are strong loss-of-function or null alleles, causing severe dendrite branching defects and are included for comparisons. 1-day-old adult animals were imaged for all groups. Arrowheads: dendrites failed to grow high-ordered or ectopic branches (in *eff-1* group). Scale bars: 10 μm. **e** Quantifications of the number of secondary (2°), tertiary (3°) and quaternary (4°) branches in the 100 μm region anterior to the PVD cell body in different genetic backgrounds, or ectopic dendritic branches in a lateral region 100 μm anterior to the PVD cell body (between the primary dendrites and the tertiary dendrites) in *eff-1* as shown in (**d**). All values are presented as mean ± s.e.m. n⩾19 worms. Ns: not significant. * $p < 0.05$; ** $p < 0.01$; **** $p < 0.0001$ (one-way ANOVA with the Tukey correction). Source data are provided as a Source Data file.

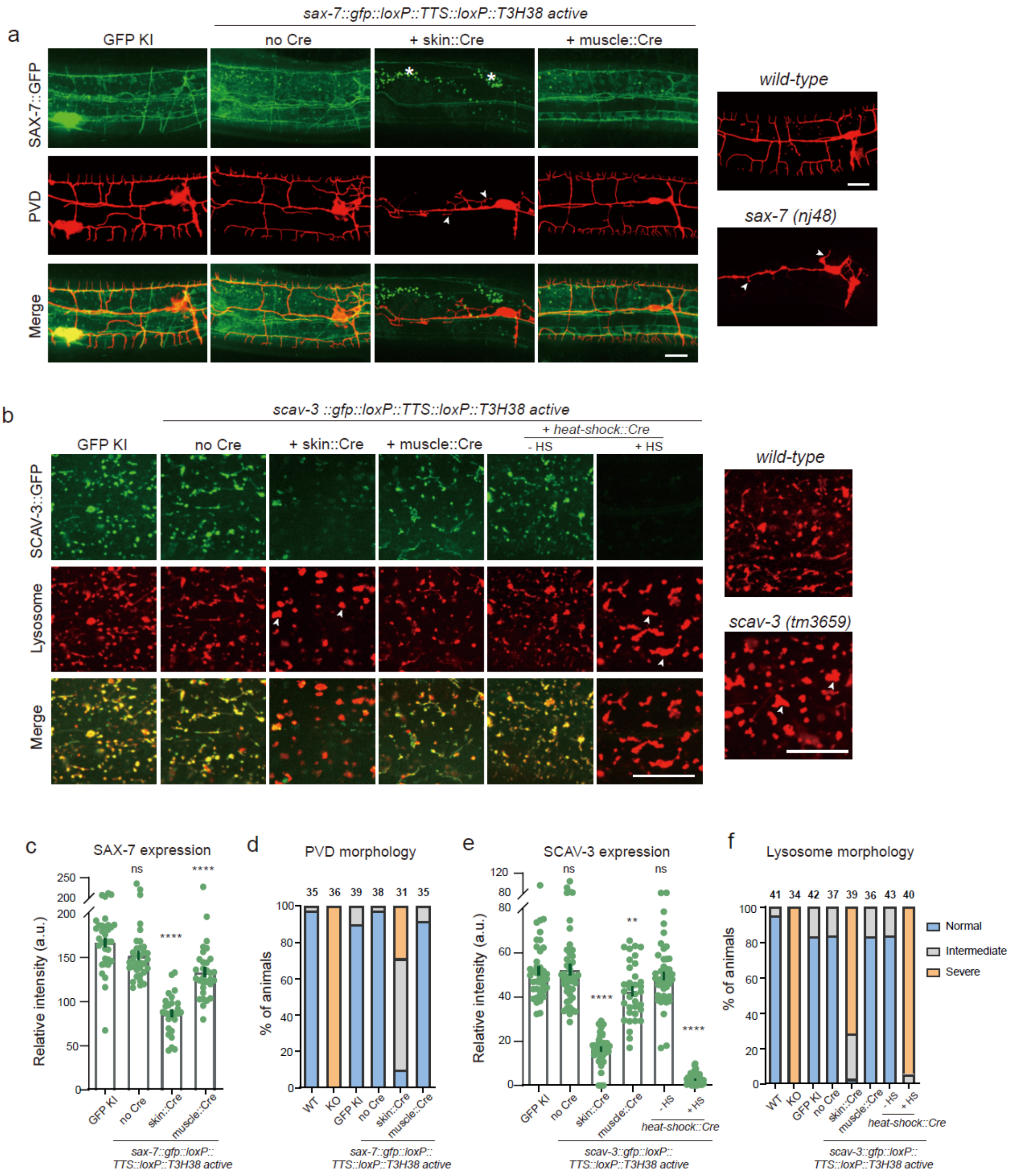
a
sax-7::gfp::loxP::TTS::loxP::T3H38 active
GFP KI
no Cre
+ skin::Cre
+ muscle::Cre
SAX-7::GFP
PVD
Merge
wild-type
sax-7 (nj48)
b
scav-3 ::gfp::loxP::TTS::loxP::T3H38 active
GFP KI
no Cre
+ skin::Cre
+ muscle::Cre
+ heat-shock::Cre
- HS
+ HS
SCAV-3::GFP
Lysosome
Merge
wild-type
scav-3 (tm3659)
c
SAX-7 expression
Relative intensity (a.u.)
ns
****
****
GFP KI
no Cre
skin::Cre
muscle::Cre
sax-7::gfp::loxP::
TTS::loxP::T3H38 active
d
PVD morphology
% of animals
35 36 39 38 31 35
WT
KO
GFP KI
no Cre
skin::Cre
muscle::Cre
sax-7::gfp::loxP::
TTS::loxP::T3H38 active
e
SCAV-3 expression
Relative intensity (a.u.)
ns
****
**
ns
****
GFP KI
no Cre
skin::Cre
muscle::Cre
- HS
+ HS
heat-shock::Cre
scav-3::gfp::loxP::
TTS::loxP::T3H38 active
f
Lysosome morphology
% of animals
41 34 42 37 39 36 43 40
Normal
Intermediate
Severe
WT
KO
GFP KI
no Cre
skin::Cre
muscle::Cre
- HS
+ HS
heat-shock::Cre
scav-3::gfp::loxP::
TTS::loxP::T3H38 active

Supplementary Fig. 3

Conditional inactivation of *scav-3* and *sax-7* via Ribo-Off.

a Confocal fluorescent images to show SAX-7::GFP expression and PVD morphologies in different genetic backgrounds. All animals were imaged at 2-day-old adults. Arrowheads: defective dendrite branching. Asterisks: gut autofluorescence. Scale bars: 20 μm. **b** Confocal fluorescent images showing SCAV-3::GFP expression and the morphology of lysosomes in various genetic backgrounds as indicated. Cre expression was temporally controlled by a heat-shock promoter and spatially controlled by tissue-specific promoters, respectively. All animals were imaged at day 2 of adulthood. Arrowheads: enlarged lysosomes. Scale bars: 20 μm. **c** Relative SAX-7::GFP expression under different conditions as show in (**a**). Each dot represents a single worm, $n \geqslant 28$. All values are presented as mean ± s.e.m. **d** Quantifications of dendrite morphogenesis under different conditions. Sample size is indicated above each column. **e** Relative SCAV-3::GFP expression under various conditions as show in (**d**). Each dot represents a single worm, $n \geqslant 35$. All values are presented as mean ± s.e.m.. **f** Quantifications of lysosome morphology in different genetic backgrounds as indicated in (**b**). Sample size is indicated for each column. For (**c**) and (**e**): Ns: not significant. ** $p < 0.01$. **** $p < 0.0001$ (one-way ANOVA with the Tukey correction). Source data are provided as a Source Data file.

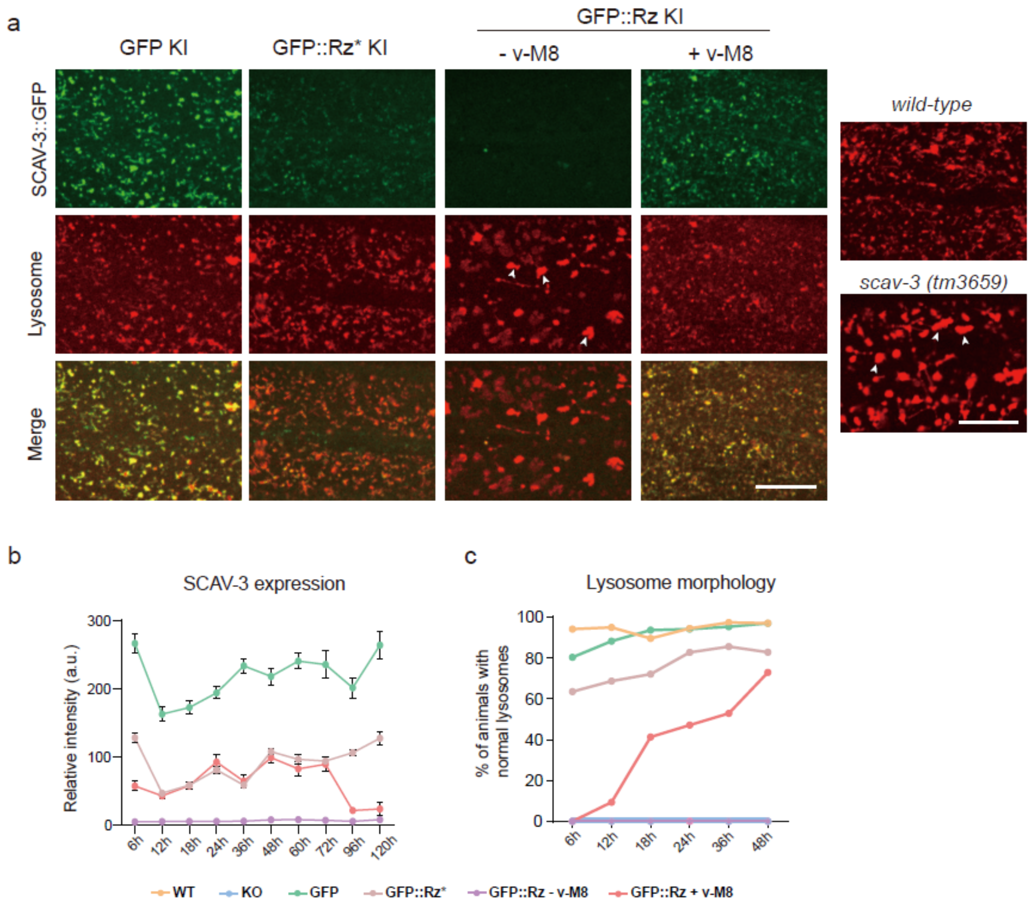

Supplementary Fig. 4

Time-course analysis of endogenous *scav-3* on-switch induced by morpholinos.

a Confocal fluorescent images of endogenous *scav-3* carrying GFP, GFP::Rz*, GFP::Rz and GFP::Rz regulated by V-M8. Arrowheads: enlarged lysosomes. Scale bar: 20 µm. **b** Relative SCAV-3::GFP expression was measured at the indicated time points (hours after v-M8 morpholino or M9 injection). All values are presented as presented as mean ± s.e.m. n≥20 worms. **c** Line plots to show the proportion of animals containing lysosomes with normal morphology. For each condition, n≥28 worms. Source data are provided as a Source Data file.

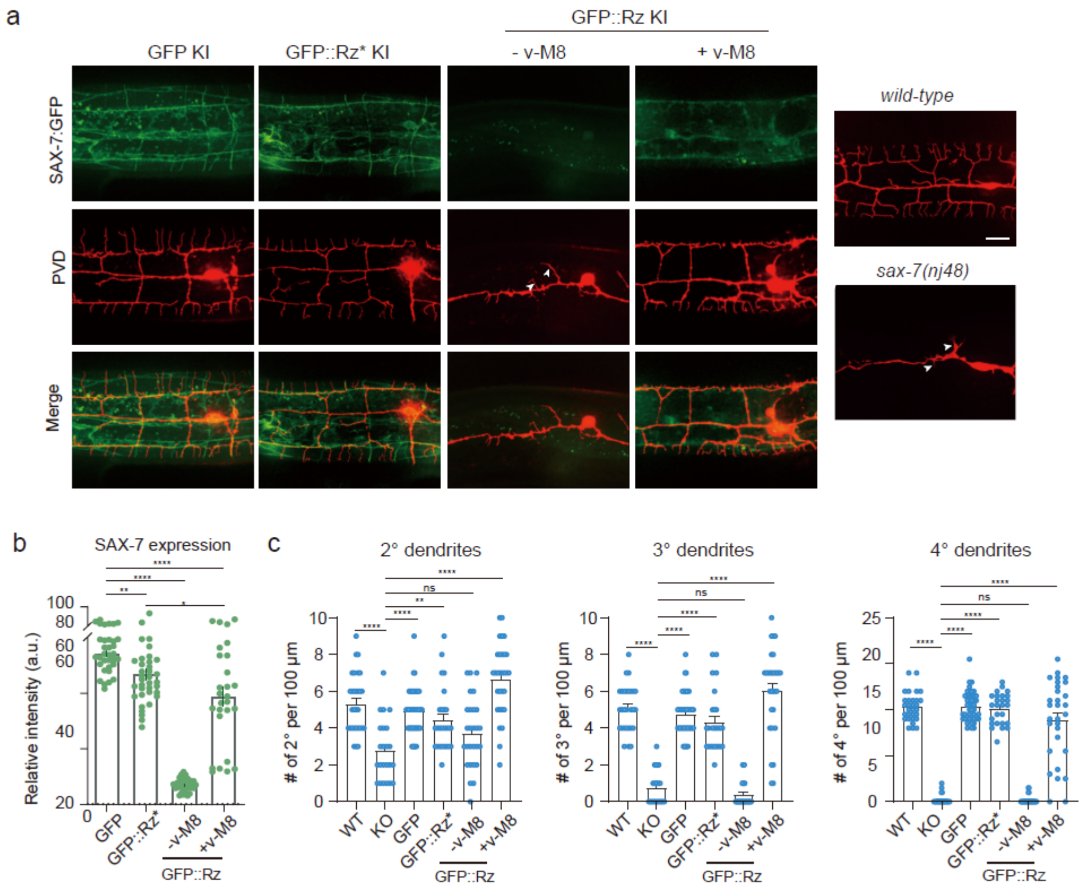

Supplementary Fig. 5

On-switch of endogenous *sax-7* expression induced by morpholinos.

a Confocal fluorescent images of endogenous *sax-7* carrying GFP, GFP::Rz*, GFP::Rz and GFP::Rz regulated by V-M8. Arrowheads: defective dendrite branching. Scale bars: 20 μm. **b** Relative expression of SAX-7::GFP quantified by fluorescent imaging under different conditions as show in (**a**). Each dot represents a single worm, n≥27. All values are presented as mean ± s.e.m. **c** Quantifications of number of secondary (2°), tertiary (3°) and quaternary (4°) branches in the 100 μm region anterior to the PVD cell body. n≥23 worms. Ns: not significant. * $p < 0.05$.** $p < 0.01$. **** $p < 0.0001$ (one-way ANOVA with the Tukey correction). Source data are provided as a Source Data file.

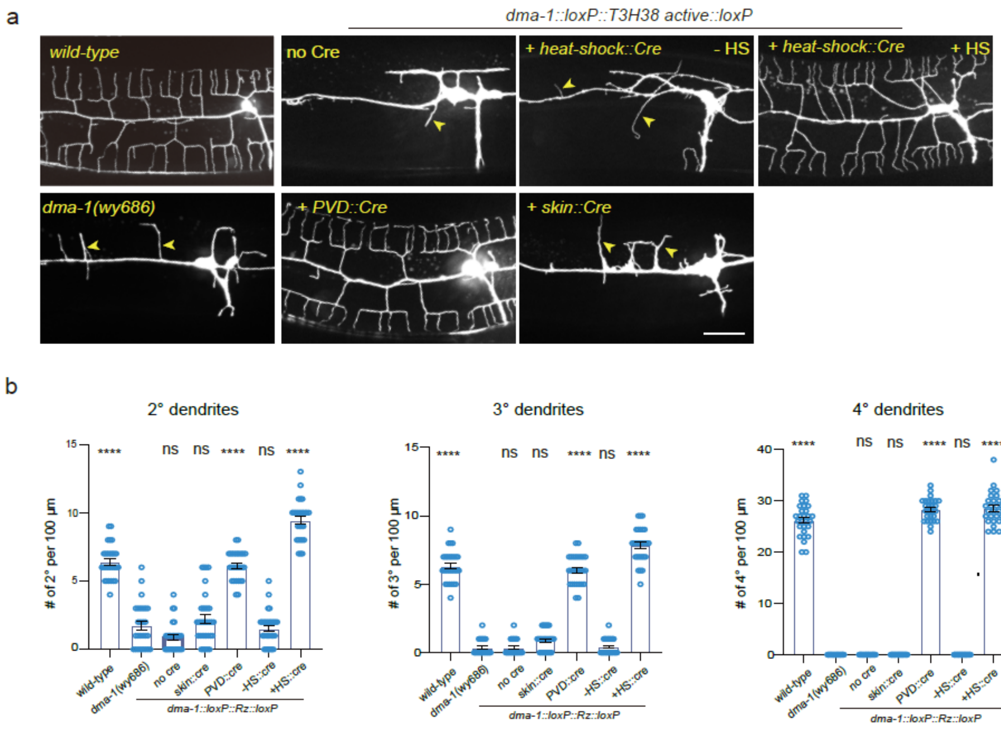

Supplementary Fig. 6

The Ribo-On system turns on endogenous *dma-1* gene in space and time.

a Confocal fluorescent images to show dendrite morphogenesis indicated by *PVD::myr-gfp* in indicated genetic backgrounds. Cre expression was spatially controlled by tissue-specific promoters and temporally controlled by a heat-shock promoter, respectively. All adult animals were imaged at day 1 of adulthood. Arrowheads: defective dendrite branching. Scale bars: 20 μm. **b** Quantifications of number of secondary (2°), tertiary (3°) and quaternary (4°) branches in the 100 μm region anterior to the PVD cell body. Ns: not significant. **** $p < 0.0001$ (one-way ANOVA with the Tukey correction). n≥15 animals for each column. Source data are provided as a Source Data file.

Supplementary Table 1. Strains used in this study

Strain name	Detailed information	Notes
SHX377	*zjuSi47[Pcol-19>mito::mKate2]*	Fig 1c, Fig 2a, Fig 3b, Fig 5b and Fig 6b
GSW7343	*argn-1(zac427); zjuSi47[Pcol-19>mito::mKate2]*	Fig 1c, Fig 2a, Fig 3b, Fig 5b and Fig 6b
GSW7284	*argn-1(zac343[argn-1::gfp]); zjuSi47[Pcol-19>mito::mKate2]*	Fig 1c, Fig 3b, Fig 5b and Fig 6b
GSW7285	*argn-1(zac392[argn-1::gfp::T3H38 active]); zjuSi47[Pcol-19>mito::mKate2]*	Fig 1c, Fig 2a and Fig 5e
GSW7286	*argn-1(zac393[argn-1::gfp::T3H38 inactive]); zjuSi47[Pcol-19>mito::mKate2]*	Fig 1c and Fig 5e
GSW7400	*zacSi13(Psemo-1>ctns-1-mcherry)*	Fig 1c and Fig S4a
GSW7431	*scav-3(tm3659); zacSi13(Psemo-1>ctns-1-mcherry)*	Fig 1c, Fig S3d and Fig S4a
GSW7434	*scav-3(zac389[scav-3::gfp]); zacSi13(Psemo-1>ctns-1-mcherry)*	Fig 1c, Fig S3d and Fig S4a
GSW7432	*scav-3(zac386[scav-3::gfp::T3H38 active]); zacSi13(Psemo-1>ctns-1-mcherry)*	Fig 1c, Fig 2a and Fig S4a
GSW7433	*scav-3(zac387[scav-3::gfp::T3H38 inactive]); zacSi13(Psemo-1>ctns-1-mcherry)*	Fig 1c and Fig S4a
TV15919	*wyIs587[ser2prom3>myr-mcherry]*	Fig 1c, Fig S3a and Fig S5a
GSW7234	*sax-7(nj48); wyIs587[ser2prom3>myr-mcherry]*	Fig 1c, Fig S3a and Fig S5a
GSW7020	*sax-7(zac311[sax-7::gfp]; wyIs587[ser2prom3>myr-mcherry]*	Fig 1c and Fig S5a
GSW7124	*sax-7(zac349[sax-7::gfp::T3H38 active]; wyIs587[ser2prom3>myr-mcherry]*	Fig 1c and Fig S5a
GSW7125	*sax-7(zac350[sax-7::gfp::T3H38 inactive]; wyIs587[ser2prom3>myr-mcherry]*	Fig 1c and Fig S5a
GSW6981	*drp-1(tm1108); zjuSi48[Pcol-19>tomm-20::gfp]*	Fig 2a and Fig S2b
GSW567	*drp-1(zac567[drp-1::loxp::T3H38 active::loxp)*	Fig 2a and Fig S2b
GSW481	*scav-3(zac481[scav-3::loxp::T3H38::loxp]); qxIs257[Pced-1::nuc-1::mCherry]*	Fig 2a
TV17248	*sax-7(nj48); wyIs592[ser2prom3>myr-gfp]*	Fig 2a
GSW479	*sax-7(zac479[sax-7::loxp::T3H38::loxp]); wyIs592[ser2prom3>myr-gfp]*	Fig 2a
GSW5004	*eff-1(hy21); wyIs592[ser2prom3>myr-gfp]*	Fig 2a and Fig S2d
GSW8155	*eff-1(zac527[eff-1b::loxp::T3H38 acitive]); hrtIs3[Pdes-2::myri-gfp]*	Fig 2a and Fig S2d

TV15911	*wyIs592[ser2prom3>myr-gfp]*	Fig 2a, Fig S2d and S6a
TV19647	*dma-1(wy686);wyIs592[ser2prom3>myr-gfp]*	Fig 2a, Fig S2d and S6a
GSW490	*dma-1(zac490[dma-1::loxp::T3H38::loxp]);wyIs592[ser2prom3>myr-gfp]*	Fig 2a, Fig S2d and S6a
XW5399	*qxIs257[Pced-1::nuc-1::mCherry]*	Fig2a and Fig S3d
GSW7782	*scav-3(tm3659); qxIs257[Pced-1::nuc-1::mCherry]*	Fig2a and Fig S3d
GSW467	*argn-1(zac467[argn-1::gfp::loxp::let-858 3' UTR::loxp:: T3H38 active]); zjuSi47[Pcol-19>mito::mKate2]*	Fig 3b
GSW7738	*argn-1(zac467[argn-1::gfp::loxp::let-858 3' UTR::loxp:: T3H38 active]); tmIs1028[Pdpy-7>NLS::Cre]; zjuSi47[Pcol-19>mito::mKate2]*	Fig 3b
GSW7739	*argn-1(zac467[argn-1::gfp::loxp::let-858 3' UTR::loxp:: T3H38 active]); tmIs1055[Phsp-16.2>Cre]; zjuSi47[Pcol-19>mito::mKate2]*	Fig 3b
GSW7740	*argn-1(zac467[argn-1::gfp::loxp::let-858 3' UTR::loxp:: T3H38 active]); tmIs1058[Pmyo-3>NLS::Cre]; zjuSi47[Pcol-19>mito::mKate2]*	Fig 3b
GSW476	*bicd-1(zac476[bicd-1::loxp::let-858 3' UTR::loxp::T3H38]);wyIs592[ser2prom3>myr-gfp]*	Fig 4b
GSW7974	*bicd-1(zac476[bicd-1::loxp::let-858 3' UTR::loxp::T3H38]);hrtSi27(des-2p::Cre);wyIs592[ser2prom3>myr-gfp]*	Fig 4b
GSW7845	*bicd-1(zac476[bicd-1::loxp::let-858 3' UTR::loxp::T3H38]);tmIs1087(myo-3p::Cre);zacEx1846[ser2prom3>myr-gfp]*	Fig 4b
GSW7820	*bicd-1(zac476[bicd-1::loxp::let-858 3' UTR::loxp::T3H38]);wyIs592[ser2prom3>myr-gfp];tmIs1027(dpy-7p::Cre)*	Fig 4b
GSW5014	*bicd-1 (zac51); wyIs594[ser2prom3>myr-gfp]*	Fig 4b
GSW475	*argn-1(zac475[argn-1::gfp::loxp::T3H38::loxp]);zjuSi47[Pcol-19>mito::mKate2]*	Fig 6b
GSW7825	*argn-1(zac475[argn-1::gfp::loxp::T3H38::loxp]);tmIs1028(dpy-7p::Cre);zjuSi47[Pcol-19>mito::mKate2]*	Fig 6b
GSW7827	*argn-1(zac475[argn-1::gfp::loxp::T3H38::loxp]);tmIs1055(hsp16.2p::Cre) ;zjuSi47[Pcol-19>mito::mKate2]*	Fig 6b
GSW7910	*argn-1(zac475[argn-1::gfp::loxp::T3H38::loxp]);tmIs1058(myo-3p::Cre);zjuSi47[Pcol-19>mito::mKate2]*	Fig 6b

FU5113	*dhgd-1(tm6671); qyIs157[Psemo-1>mito-gfp]*	Fig S2b
GSW565	*dhgd-1(zac565[dhgd-1::loxp::T3H38 active::loxp]); zjuSi48[Pcol-19>tomm-20::gfp]*	Fig S2b
FU5195	*hphd-1(ok3580); qyIs157[Psemo-1>mito-gfp]*	Fig S2b
GSW562	*hphd-1(zac564[hphd-1::loxp::T3H38 active::loxp]); zjuSi48[Pcol-19>tomm-20::gfp]*	Fig S2b
GSW5007	*unc-86(e1416);wyIs587[ser2prom3>myr-mcherry]*	Fig S2d
GSW587	*unc-86(zac587[unc-86::loxp::T3H38::loxp]);wyIs592[ser2prom3>myr-gfp]*	Fig S2d
GSW5005	*mec-3(e1338);wyIs592[ser2prom3>myr- gfp]*	Fig S2d
GSW572	*mec-3(zac572[mec-3::loxp::T3H38::loxp]);wyIs592[ser2prom3>myr-gfp]*	Fig S2d
GSW8302	*hpo-30(ok2047); wyIs592[ser2prom3>myr-gfp]*	Fig S2d
GSW561	*hpo-30(zac561[hpo-30::loxp::T3H38 active::loxp]); wyIs592[ser2prom3>myr-gfp]*	Fig S2d
GSW5045	*kpc-1(gk8);wyIs592[ser2prom3>myr- gfp]*	Fig S2d
GSW7432	*kpc-1(zac571[kpc-1::loxp::T3H38::loxp]);wyIs592[ser2prom3>myr-gfp]*	Fig S2d
TV19210	*rab-10(ok1494); wyIs592[ser2prom3>myr- gfp]*	Fig S2d
GSW586	*rab-10(zac586[rab-10::loxp::T3H38::loxp]);wyIs592[ser2prom3>myr-gfp]*	Fig S2d
TV16271	*mnr-1(wy758); wyIs592[ser2prom3>myr- gfp]*	Fig S2d
GSW573	*mnr-1(zac573[mnr-1::loxp::T3H38::loxp]);wyIs592[ser2prom3>myr-gfp]*	Fig S2d
TV17245	*lect-2(ok2617);wyIs592[ser2prom3>myr-gfp]*	Fig S2d
GSW474	*lect-2(zac474[lect-2::loxp::T3H38::loxp]);wyIs592*	Fig S2d
GSW7783	*sax-7(zac465[sax-7::gfp::loxp::let-858 3' UTR::loxp::T3H38 active]); wyIs587[ser2prom3>myr-mcherry]*	Fig S3a
GSW7778	*sax-7(zac465[sax-7::gfp::loxp::let-858 3' UTR::loxp::T3H38 active]); tmIs1087[Pmyo-3>NLS::Cre]; zacEx1828[ser2prom3>myr-mcherry]*	Fig S3a
GSW7779	*sax-7(zac465[sax-7::gfp::loxp::let-858 3' UTR::loxp::T3H38 active]); tmIs1028[Pdpy-7>NLS::Cre]; zacEx1825[ser2prom3>myr-mcherry]*	Fig S3a
GSW7791	*scav-3(zac387[scav-3::gfp]); qxIs257[Pced-1::nuc-1::mCherry]*	Fig S3d
GSW7796	*scav-3(zac469[scav-3::gfp::loxp::let-858 3' UTR::loxp::T3H38 active]); qxIs257[Pced-1::nuc-1::mCherry]*	Fig S3d
GSW7789	*scav-3(zac469[scav-3::gfp::loxp::let-858 3' UTR::loxp::T3H38 active]); tmIs1027[Pdpy-7>NLS::Cre]; qxIs257[Pced-1::nuc-1::mCherry]*	Fig S3d

GSW7811	*scav-3(zac469[scav-3::gfp::loxp::let-858 3' UTR::loxp::T3H38 active]); tmIs1087[Pmyo-3>NLS::Cre]; qxIs257[Pced-1::nuc-1::mCherry]*	Fig S3d
GSW7790	*scav-3(zac469[scav-3::gfp::loxp::let-858 3' UTR::loxp::T3H38 active]); tmIs1054[Phsp-16.2>Cre]; qxIs257[Pced-1::nuc-1::mCherry]*	Fig S3d
GSW7839	*dma-1(zac490[dma-1::loxp::T3H38::loxp]);tmIs1028(dpy-7p::cre);zacEx1840 [ser2prom3> myr-gfp]*	Fig S6a
GSW7819	*dma-1(zac490[dma-1::loxp::T3H38::loxp]);hrtSi27(des-2p::cre);wyIs592[ser2prom3>myr-gfp]*	Fig S6a
GSW7842	*dma-1(zac490[dma-1::loxp::T3H38::loxp]);tmIs1055(hsp16.2p::cre);zacEx1843 (Ex[ser2prom3> myr- gfp])*	Fig S6a

Supplementary Table 2. Plasmids used in this study

Plasmid name	Detailed information	Notes
pDD 122	*Peft-3-Cas9-NLS-pU6-ttTi5605 sgRNA*	sg*ChrII* for making single-copy transgene
pFJ147	*ChrII-Phpy-7>ctns-1::mcherry::loxp::unc-119::loxp::mcherry::unc-54 UTR*	Repair template to generate *zacSi13* using CRISPR
pFJ121	*pU6(GB)-argn-1-sg1*	*argn-1-sg1* for CRISPR KI
pFJ152	*pU6(GB)-argn-1-sg2*	*argn-1-sg2* for CRISPR KO
pFJ153	*pU6(GB)-argn-1-sg3*	*argn-1-sg3* for CRISPR KO
pFJ154	*pU6(GB)-argn-1-sg4*	*argn-1-sg4* for CRISPR KO
pFJ123	*argn-1-gfp-loxp-unc-119-loxp donor*	Repair template to generate *argn-1(zac343)*
pFJ126	*argn-1-gfp-loxp-unc-119-loxp-T3H38 active donor*	Repair template to generate *argn-1(zac392)*
pFJ127	*argn-1-gfp-loxp-unc-119-loxp-T3H38 inactive donor*	Repair template to generate *argn-1(zac393)*
pFJ184	*argn-1-gfp-loxp-let 858-loxp-T3H38 active donor*	Repair template to generate *argn-1(zac467)*
pWJ405	*argn-1::gfp::loxp::T3H38::loxp donor*	Repair template to generate *argn-1(zac475)*
pFJ119	*pU6(GB)-scav-3-sg1*	*scav-3-sg1* for CRISPR KI
pFJ120	*pU6(GB)-scav-3-sg2*	*scav-3-sg2* for CRISPR KI
pFJ122	*scav-3-gfp-loxp-unc-119-loxp donor*	Repair template to generate *scav-3(zac389)*
pFJ124	*scav-3-gfp-loxp-unc-119-loxp-T3H38 active donor*	Repair template to generate *scav-3(zac389)*
pFJ125	*scav-3-gfp-loxp-unc-119-loxp-T3H38 inactive donor*	Repair template to generate *scav-3(zac386)*
pFJ185	*scav-3-gfp-loxp-let 858-loxp-T3H38 active donor*	Repair template to generate *scav-3(zac469)*
pFJ59	*pU6(GB)-sax-7-sg2*	*sax-7-sg2* for CRISPR KI
pFJ60	*pU6(GB)-sax-7-sg3*	*sax-7-sg3* for CRISPR KI
pFJ94	*sax-7-gfp-loxp-unc-119-loxp donor*	Repair template to generate *sax-7(zac311)*
pFJ117	*sax-7-gfp-loxp-loxp-unc-119-loxp-T3H38 active-loxp donor*	Repair template to generate *sax-7(zac349)*
pFJ118	*sax-7-gfp-loxp-loxp-unc-119-loxp-T3H38 inactive-loxp donor*	Repair template to generate *sax-7(zac350)*
pFJ183	*sax-7-gfp-loxp-let 858-loxp-T3H38 active donor*	Repair template to generate *sax-7(zac465)*
pWJ396	*pU6 (GB)- dma-1-sg1*	To generate *dma-1(zac490)*

pWJ397	*pU6 (GB)- dma-1-sg2*	To generate *dma-1(zac490)*
pWJ398	*dma-1::loxp::T3H38::loxp donor*	Repair template to generate *dma-1(zac490)*
pWJ399	*pU6 (GB)- lect-2-sg1*	To generate *lect-2(zac474)*
pWJ400	*pU6 (GB)- lect-2-sg2*	To generate *lect-2(zac474)*
pWJ401	*lect-2::loxp::T3H38::loxp donor*	Repair template to generate *lect-2(zac474)*
pWJ387	*pU6 (GB)- bicd-1-sg1*	To generate *bicd-1(zac476)*
pWJ388	*pU6 (GB)- bicd-1-sg2*	To generate *bicd-1(zac476)*
pWJ389	*pU6 (GB)- bicd-1-sg3*	To generate *bicd-1(zac476)*
pWJ390	*bicd-1::loxp::let-858 stop::loxp::T3H38 donor*	Repair template to generate *bicd-1(zac476)*
pFJ246	*pU6 (GB)-eff-1b-sg1*	To generate *eff-1(zac527)*
pFJ247	*pU6 (GB)-eff-1b-sg2*	To generate *eff-1(zac527)*
pFJ249	*eff-1b::loxp::T3H38 active::loxp donor*	Repair template to generate *eff-1(zac527)*
pFJ76	*pU6 (GB)-drp-1-sg1*	To generate *drp-1(zac567)*
pFJ301	*drp-1::loxp::T3H38 active::loxp donor*	Repair template to generate *drp-1(zac567)*
pFJ56	*pU6 (GB)-hpo-30-sg1*	To generate *hpo-30(zac561)*
pFJ299	*hpo-30::loxp::T3H38 active::loxp donor*	Repair template to generate *hpo-30(zac561)*
pFJ303	*pU6 (GB)-hphd-1-sg1*	To generate *hphd-1(zac564)*
pFJ304	*pU6 (GB)-hphd-1-sg2*	To generate *hphd-1(zac564)*
pFJ298	*hphd-1::loxp::T3H38 active::loxp donor*	Repair template to generate *hphd-1(zac564)*
pFJ305	*pU6 (GB)-dhgd-1-sg1*	To generate *dhgd-1(zac565)*
pFJ306	*pU6 (GB)-dhgd-1-sg2*	To generate *dhgd-1(zac565)*
pFJ307	*pU6 (GB)-dhgd-1-sg3*	To generate *dhgd-1(zac565)*
pFJ300	*dhgd-1::loxp::T3H38 active::loxp donor*	Repair template to generate *dhgd-1(zac565)*
pFJ314	*T7>argn-1 cDNA*	To produce *argn-1* dsRNA in HT115
pFJ315	*T7>sax-7 cDNA*	To produce *sax-7* dsRNA in HT115
pWJ505	*pU6 (GB)- unc-86-sg1*	To generate *unc-86(zac587)*
pWJ506	*pU6 (GB)- unc-86-sg2*	To generate *unc-86(zac587)*

pWJ507	*pU6 (GB)- unc-86-sg3*	To generate *unc-86(zac587)*
pWJ504	*unc-86::loxp::T3H38::loxp donor*	Repair template to generate *unc-86(zac587)*
pWJ508	*pU6 (GB)- rab-10-sg1*	To generate *rab-10(zac586)*
pWJ509	*pU6 (GB)- rab-10-sg2*	To generate *rab-10(zac586)*
pWJ510	*pU6 (GB)- rab-10-sg3*	To generate *rab-10(zac586)*
pWJ511	*rab-10::loxp::T3H38::loxp donor*	Repair template to generate *rab-10(zac586)*
pWJ512	*pU6 (GB)- mnr-1-sg1*	To generate *mnr-1(zac573)*
pWJ513	*pU6 (GB)- mnr-1-sg2*	To generate *mnr-1(zac573)*
pWJ514	*pU6 (GB)- mnr-1-sg3*	To generate *mnr-1(zac573)*
pWJ515	*mnr-1::loxp::T3H38::loxp donor*	Repair template to generate *mnr-1(zac573)*
pWJ516	*pU6 (GB)- mec-3-sg1*	To generate *mec-3(zac572)*
pWJ517	*pU6 (GB)- mec-3-sg2*	To generate *mec-3(zac572)*
pWJ518	*mec-3::loxp::T3H38::loxp donor*	Repair template to generate *mec-3(zac572)*
pWJ519	*pU6 (GB)- kpc-1-sg1*	To generate *kpc-1(zac571)*
pWJ520	*pU6 (GB)- kpc-1-sg2*	To generate *kpc-1(zac571)*
pWJ521	*pU6 (GB)- kpc-1-sg3*	To generate *kpc-1(zac571)*
pWJ522	*kpc-1::loxp::T3H38::loxp donor*	Repair template to generate *kpc-1(zac571)*

Supplementary Table 3. Primers used in this study

Name	Sequence	Note	Used in which plasmid or fusion PCR product
oFJ511	AGCCAGGAACTTCGGCTCAG	qPCR for *ama-1*, S	N/A
oFJ512	CATAAGTCGGCGAGCTTG	qPCR for *ama-1*, AS	N/A
oFJ517	GATGCACATATGGATGCCCA	qPCR for *argn-1*, S	N/A
oFJ518	GTGAAAATCGGAGATCGGCT	qPCR for *argn-1*, AS	N/A
oFJ539	ACACAAAAGATGTCGCTGTG	qPCR for *scav-3*, S	N/A
oFJ540	CGCGGCATTCCATTCTTATT	qPCR for *scav-3*, AS	N/A
oFJ532	GCTGATGATGGACTTACCGT	qPCR for *sax-7*, S	N/A
oFJ533	CAGACAACGTTTTCGTCGTT	qPCR for *sax-7*, AS	N/A
sgRNA-Rev	/5' Phos/ **c**aagacatctcgcaatagg aggtg	Antisense primer to make sgRNA. Note that a "C" was added as the U6 promoter requires a "G" base at the transcription start site	All sgRNA plasmids.
oFJ340	AGACAAGTCATCAGAGCTATGTTTAAGAGCTATGCTGGAAACAGC	Sense primer to make gRNA for CRISPR KO of *argn-1*. Target DNA sequence was underlined.	pFJ152
oFJ341	ATCATAGAAGAGGTGAACACGTTTAAGAGCTATGCTGGAAACAGC	Sense primer to make gRNA for CRISPR KO of *argn-1*. Target DNA sequence was underlined.	pFJ153
oFJ342	ATAGTTGTGCAATCGGAACGGTTTAAGAGCTATGCTGGAAACAGC	Sense primer to make gRNA for CRISPR KO of *argn-1*. Target DNA sequence was underlined.	pFJ154
oFJ246	TGCACAACGCGTGAGCACGTGTTTAAGAGCTATGCTGGAAACAGC	Sense primer to make sgRNA for CRISPR KI of *argn-1*. Target DNA sequence was underlined.	pFJ121
oFJ244	GCAAGACGAGAACGCAGTTTGTTTAAGAGCTATGCTGGAAACAGC	Sense primer to make sgRNA for CRISPR KI of *scav-3*. Target DNA sequence was underlined.	pFJ119
oFJ245	ACTGCGTTCTCGTCTTGCGCGTTTAAGAGCTATGCTGGAAACAGC	Sense primer to make sgRNA for CRISPR KI of *scav-3*. Target DNA sequence was underlined.	pFJ120
oFJ118	GTCGACGTTGATCCTTTCTGTTTAAGAGCTATGCTGGAAACAGC	Sense primer to make sgRNA for CRISPR KI of *sax-7*. Target DNA sequence was underlined.	pFJ59
oFJ119	TCGACGTTGATCCTTTCTCGTTTAAGAGCTATGCTGGAAACAGC	Sense primer to make sgRNA for CRISPR KI of *sax-7*. Target DNA sequence was underlined.	pFJ60
oWJ663	aacctggatcatcctatttGTTTAAGAGCTATGCTGGAAACAGC	Sense primer to make sgRNA for CRISPR KI of *dma-1*. Target DNA	pWJ396

		sequence was underlined.	
oWJ664	gatatttaaatgccaaaatGTTTAAGAGCTATGCTGGAAACAGC	Sense primer to make sgRNA for CRISPR KI of *dma-1*. Target DNA sequence was underlined.	pWJ397
oWJ669	aaggaactgtttagaatacGTTTAAGAGCTATGCTGGAAACAGC	Sense primer to make sgRNA for CRISPR KI of *lect-2*. Target DNA sequence was underlined.	pWJ399
oWJ670	tttagaaTactggaaaGttGTTTAAGAGCTATGCTGGAAACAGC	Sense primer to make sgRNA for CRISPR KI of *lect-2*. Target DNA sequence was underlined.	pWJ400
oWJ647	cgaggggagggccacgcagGTTTAAGAGCTATGCTGGAAACAGC	Sense primer to make sgRNA for CRISPR KI of *bicd-1*. Target DNA sequence was underlined.	pWJ387
oWJ648	tcgaggggagggccacgcaGTTTAAGAGCTATGCTGGAAACAGC	Sense primer to make sgRNA for CRISPR KI of *bicd-1*. Target DNA sequence was underlined.	pWJ388
oWJ649	gctgattgtctcccctgcgGTTTAAGAGCTATGCTGGAAACAGC	Sense primer to make sgRNA for CRISPR KI of *bicd-1*. Target DNA sequence was underlined.	pWJ389
oFJ112	catcgtcaatcactacaaaGTTTAAGAGCTATGCTGGAAACAGC	Sense primer to make sgRNA for CRISPR KI of *hpo-30*. Target DNA sequence was underlined.	pFJ56
oFJ141	aagtgagagaaacacaagttGTTTAAGAGCTATGCTGGAAACAGC	Sense primer to make sgRNA for CRISPR KI of *argn-1*. Target DNA sequence was underlined.	pFJ76
oFJ544	tttgtctcagaatccgaagaGTTTAAGAGCTATGCTGGAAACAGC	Sense primer to make sgRNA for CRISPR KI of *eff-1*. Target DNA sequence was underlined.	pFJ246
oFJ545	actacttcttgcaccatcttGTTTAAGAGCTATGCTGGAAACAGC	Sense primer to make sgRNA for CRISPR KI of *eff-1*. Target DNA sequence was underlined.	pFJ247
oFJ686	tctgtacgagaagtcccttaGTTTAAGAGCTATGCTGGAAACAGC	Sense primer to make sgRNA for CRISPR KI of *hphd-1*. Target DNA sequence was underlined.	pFJ303
oFJ687	aaatgactaataaaccgtaaGTTTAAGAGCTATGCTGGAAACAGC	Sense primer to make sgRNA for CRISPR KI of *hphd-1*. Target DNA sequence was underlined.	pFJ304
oFJ688	tagaattatatcgttttgtaGTTTAAGAGCTATGCTGGAAACAGC	Sense primer to make sgRNA for CRISPR KI of *dhgd-1*. Target DNA sequence was underlined.	pFJ305
oFJ689	agaacattttcgacccaaatGTTTAAGAGCTATGCTGGAAACAGC	Sense primer to make sgRNA for CRISPR KI of *dhgd-1*. Target DNA sequence was underlined.	pFJ306
oFJ670	tacggattgagaatcccattGTTTAAGAG	Sense primer to make sgRNA for	pFJ307

	CTATGCTGGAAACAGC	CRISPR KI of *dhgd-1*. Target DNA sequence was underlined.	
oWJ959	caacatacaatgggctaccGTTTAAGAG CTATGCTGGAAACAGC	Sense primer to make sgRNA for CRISPR KI of *unc-86*. Target DNA sequence was underlined.	pWJ505
oWJ960	gaaactaatcaaagaatccGTTTAAGAG CTATGCTGGAAACAGC	Sense primer to make sgRNA for CRISPR KI of *unc-86*. Target DNA sequence was underlined.	pWJ506
oWJ961	gggtcagacaacatacaatGTTTAAGAG CTATGCTGGAAACAGC	Sense primer to make sgRNA for CRISPR KI of *unc-86*. Target DNA sequence was underlined.	pWJ507
oWJ966	agaggcagagtagcagtggGTTTAAGA GCTATGCTGGAAACAGC	Sense primer to make sgRNA for CRISPR KI of *rab-10*. Target DNA sequence was underlined.	pWJ508
oWJ967	cacagaggcagagtagcagGTTTAAGA GCTATGCTGGAAACAGC	Sense primer to make sgRNA for CRISPR KI of *rab-10*. Target DNA sequence was underlined.	pWJ509
oWJ968	gaatccagtgcaaccacagGTTTAAGAG CTATGCTGGAAACAGC	Sense primer to make sgRNA for CRISPR KI of *rab-10*. Target DNA sequence was underlined.	pWJ510
oWJ973	cttaaagtcaaattaaagtGTTTAAGAGC TATGCTGGAAACAGC	Sense primer to make sgRNA for CRISPR KI of *mnr-1*. Target DNA sequence was underlined.	pWJ512
oWJ974	ttaaagtaggtagatcattGTTTAAGAGC TATGCTGGAAACAGC	Sense primer to make sgRNA for CRISPR KI of *mnr-1*. Target DNA sequence was underlined.	pWJ513
oWJ975	aggtagatcattaggagtaGTTTAAGAG CTATGCTGGAAACAGC	Sense primer to make sgRNA for CRISPR KI of *mnr-1*. Target DNA sequence was underlined.	pWJ514
oWJ980	ttttgcggaaatttcgaagGTTTAAGAGC TATGCTGGAAACAGC	Sense primer to make sgRNA for CRISPR KI of *mec-3*. Target DNA sequence was underlined.	pWJ516
oWJ981	ctgacttcaacgcgttttgGTTTAAGAGC TATGCTGGAAACAGC	Sense primer to make sgRNA for CRISPR KI of *mec-3*. Target DNA sequence was underlined.	pWJ517
oWJ986	tgaagttaatataaaatctGTTTAAGAGC TATGCTGGAAACAGC	Sense primer to make sgRNA for CRISPR KI of *kpc-1*. Target DNA sequence was underlined.	pWJ519
oWJ987	attttgagactttttgaatGTTTAAGAGCT ATGCTGGAAACAGC	Sense primer to make sgRNA for CRISPR KI of *kpc-1*. Target DNA sequence was underlined.	pWJ520
oWJ988	atgaagttaatataaaatcGTTTAAGAGC TATGCTGGAAACAGC	Sense primer to make sgRNA for CRISPR KI of *kpc-1*. Target DNA sequence was underlined.	pWJ521

Supplementary Table 4. Sequence of *loxP::Rz::loxP*

Sequence (5' to 3')
ATAACTTCGTATAGCATACATTATACGAAGTTATGGCGCCGGAATTCGAGCAAACAAACAAAgcgcgtcctggattccacttcgggtacatccagctgacgagtcccaaataggacgaaacgcgcCAAACAAACAAAGAATTCGTTCGGGTATAACTTCGTATAGCATACATTATACGAAGTTAT
(Text in lowercase is the sequence for active T3H38 ribozyme. Text marked by yellow is the *loxP* site. Underlined test is the spacer sequence between *loxP* and T3H38 ribozyme.)

Supplementary Table 5. Sequence of *loxP::let-858 3' UTR::loxP::Rz*

Sequence (5' to 3')
ATAACTTCGTATAGCATACATTATACGAAGTTATGGATGATCGACGCCAACGTCGTTGAATTTTCAAATTTTAAATACTGAATATTTGTTTTTTTTCCTATTATTTATTTATTCTCTTTGTGTTTTTTTTCTTGCTTTCTAAAAAATTAATTCAATCCAAATCTAAACATTTTTTTTTCTCTTTCCGTCTCCCAATTCGTATTCCGCTCCTCTCATCTGAACACAATGTGCAAGTTTATTTATCTTCTCGCTTTCATTTCATTAGGACGTGGGGGGAATTGGTGGAAGGGGGAAACACACAAAAGGATGATGGAAATGAAATAAGGACACACAATATGCAACAACATTCAATTCAGAAATATGGAGGAAGGTTTAAAAGAAAACATAAAAATATATAGAGGAGGAAGGAAACTAGATAACTTCGTATAGCATACATTATACGAAGTTATGGCGCCGGAATTCGAGCAAACAAACAAAgcgcgtcctggattccacttcgggtacatccagctgacgagtcccaaataggacgaaacgcgcCAAACAAACAAA
(Text in lowercase is the sequence for active T3H38 ribozyme. Text marked by yellow is the *loxP* site. Underlined test is the sequence for *let-858 3' UTR*. Text labeled by blue is the spacer sequence.)
