# Supplementary material for: Ribo-On and Ribo-Off tools using a self-cleaving ribozyme allow manipulation of endogenous gene expression in C. elegans
Source: Commun Biol. 2023 Aug 4;6:816. doi: 10.1038/s42003-023-05184-4 (PMC10403566; doi:10.1038/s42003-023-05184-4)
Supplement: Supplementary file 3 — Description of Additional Supplementary Files [file 42003_2023_5184_MOESM3_ESM.pdf]

Description of Additional Supplementary Files

File name: Supplementary Data 1

Description: The numerical source data that make up all graphs and charts.
