# Supplementary material for: Ribo-On and Ribo-Off tools using a self-cleaving ribozyme allow manipulation of endogenous gene expression in C. elegans
Source: Commun Biol. 2023 Aug 4;6:816. doi: 10.1038/s42003-023-05184-4 (PMC10403566; doi:10.1038/s42003-023-05184-4)
Supplement: Supplementary file 5 — Reporting Summary [file 42003_2023_5184_MOESM5_ESM.pdf]

nature portfolio

Corresponding author(s): Baohui Chen and Wei Zou

Last updated by author(s): Jul 12, 2023

Reporting Summary

Nature Portfolio wishes to improve the reproducibility of the work that we publish. This form provides structure for consistency and transparency in reporting. For further information on Nature Portfolio policies, see our Editorial Policies and the Editorial Policy Checklist.

Statistics

For all statistical analyses, confirm that the following items are present in the figure legend, table legend, main text, or Methods section.

n/a	Confirmed	
☐	☒	The exact sample size (*n*) for each experimental group/condition, given as a discrete number and unit of measurement
☐	☒	A statement on whether measurements were taken from distinct samples or whether the same sample was measured repeatedly
☐	☒	The statistical test(s) used AND whether they are one- or two-sided<br>*Only common tests should be described solely by name; describe more complex techniques in the Methods section.*
☒	☐	A description of all covariates tested
☒	☐	A description of any assumptions or corrections, such as tests of normality and adjustment for multiple comparisons
☐	☒	A full description of the statistical parameters including central tendency (e.g. means) or other basic estimates (e.g. regression coefficient) AND variation (e.g. standard deviation) or associated estimates of uncertainty (e.g. confidence intervals)
☐	☒	For null hypothesis testing, the test statistic (e.g. *F*, *t*, *r*) with confidence intervals, effect sizes, degrees of freedom and *P* value noted<br>*Give P values as exact values whenever suitable.*
☒	☐	For Bayesian analysis, information on the choice of priors and Markov chain Monte Carlo settings
☒	☐	For hierarchical and complex designs, identification of the appropriate level for tests and full reporting of outcomes
☒	☐	Estimates of effect sizes (e.g. Cohen's *d*, Pearson's *r*), indicating how they were calculated

Our web collection on statistics for biologists contains articles on many of the points above.

Software and code

Policy information about availability of computer code

Data collection	Image collection: OLYMPUS cellSens Dimension(version 3.1.1)
Data analysis	Image processing: OLYMPUS cellSens Dimension Desktop (version 2.3); ImageJ (version ij152-win-java8)Statistical analyses: GraphPad Prism (version 8); Microsoft Excel (version 2019); IBM SPSS Statistics 26

For manuscripts utilizing custom algorithms or software that are central to the research but not yet described in published literature, software must be made available to editors and reviewers. We strongly encourage code deposition in a community repository (e.g. GitHub). See the Nature Portfolio guidelines for submitting code & software for further information.

Data

Policy information about availability of data

All manuscripts must include a data availability statement. This statement should provide the following information, where applicable:

- Accession codes, unique identifiers, or web links for publicly available datasets
- A description of any restrictions on data availability
- For clinical datasets or third party data, please ensure that the statement adheres to our policy

The data that support this study are available from the corresponding authors upon reasonable request. Key plasmids will be deposited to Addgene. Source data are provided with this paper.

Research involving human participants, their data, or biological material

Policy information about studies with human participants or human data. See also policy information about sex, gender (identity/presentation), and sexual orientation and race, ethnicity and racism.

Reporting on sex and gender	N/A
Reporting on race, ethnicity, or other socially relevant groupings	N/A
Population characteristics	N/A
Recruitment	N/A
Ethics oversight	N/A

Note that full information on the approval of the study protocol must also be provided in the manuscript.

Field-specific reporting

Please select the one below that is the best fit for your research. If you are not sure, read the appropriate sections before making your selection.

☒ Life sciences ☐ Behavioural & social sciences ☐ Ecological, evolutionary & environmental sciences

For a reference copy of the document with all sections, see nature.com/documents/nr-reporting-summary-flat.pdf

Life sciences study design

All studies must disclose on these points even when the disclosure is negative.

Sample size	For the phenotype of mitochondria and lysosomes in wild-type and mutant animals, sample size was determined according to the published literatures (PMID: 24642473 and 31735670). For the PVD dendrite branching phenotype in wild-type and mutant animals, sample size was determined according to the published literatures (PMID: 29738713 and 36126047).
Data exclusions	No data were excluded from the analysis.
Replication	The qRT-PCR experiments were repeated for 3 times and all attempts were successful. All the other experiments were performed at least two times with similar results.
Randomization	All animals were randomly selected for imaging and quantification.
Blinding	The investigators were not blinded to group allocation during data collection and/or analysis as most of the phenotypes were of high penetrance.

Reporting for specific materials, systems and methods

We require information from authors about some types of materials, experimental systems and methods used in many studies. Here, indicate whether each material, system or method listed is relevant to your study. If you are not sure if a list item applies to your research, read the appropriate section before selecting a response.

Materials & experimental systems

n/a	Involved in the study	
☒	☐	Antibodies
☒	☐	Eukaryotic cell lines
☒	☐	Palaeontology and archaeology
☐	☒	Animals and other organisms
☒	☐	Clinical data
☒	☐	Dual use research of concern
☒	☐	Plants

Methods

n/a	Involved in the study	
☒	☐	ChIP-seq
☒	☐	Flow cytometry
☒	☐	MRI-based neuroimaging

Animals and other research organisms

Policy information about studies involving animals; ARRIVE guidelines recommended for reporting animal research, and Sex and Gender in Research

Laboratory animals	Multiple C. elegans strains were generated and used in this study: SHX377, GSW7343, GSW7284, GSW7285, GSW7286, GSW7400, GSW7431, GSW7434, GSW7432, GSW7433, TV15919, GSW7234, GSW7020, GSW7124, GSW7125, GSW6981, GSW567, GSW481, TV17248, GSW479, GSW5004, GSW8155, TV15911, TV19647, GSW490, XW5399, GSW7782, GSW467, GSW7738, GSW7739, GSW7740, GSW476, GSW7974, GSW7845, GSW7820, GSW5014, GSW475, GSW7825, GSW7827, GSW7910, FU5113, GSW565, FU5195, GSW562, GSW8302, GSW561, TV17245, GSW474, GSW5007, GSW587, GSW5005, GSW572, GSW5045, GSW7432, TV19210, GSW586, TV16271, GSW573, GSW7783, GSW7778, GSW7779, GSW7789, GSW7790, GSW7791, GSW7796, GSW7811, GSW7839, GSW7842, GSW7819. For the detailed strain information, see Supplementary Table 1. 1-day-old to 3-day-old hermaphrodites were imaged and quantified in this study.
Wild animals	This study did not involve wild animals.
Reporting on sex	Hermaphrodites were imaged and quantified for this study. Male C. elegans were only used for mating to generate strains.
Field-collected samples	This study did not involve samples collected from the field.
Ethics oversight	No ethical approval was required as we used C. elegans in this study.

Note that full information on the approval of the study protocol must also be provided in the manuscript.
